# Isolation of Secondary Metabolites from *Protea venus* and Evaluation of Their Antioxidant Activity and Effects Under Glucolipotoxic Stress: In Silico and In Vitro Studies

**DOI:** 10.3390/plants15132072

**Published:** 2026-07-03

**Authors:** Kadidiatou O. Ndjoubi, Nonhlakanipho F. Sangweni, Pritika Ramharack, Rabia Johnson, Jeanine L. Marnewick, Ahmed A. Hussein

**Affiliations:** 1 Chemistry Department, Cape Peninsula University of Technology, Symphony Road, Bellville, Cape Town 7535, South Africa; dickakadi@yahoo.fr; 2 Biomedical Research and Innovation Platform (BRIP), South African Medical Research Council (SAMRC), Tygerberg, Cape Town 7505, South Africa; nonhlakanipho.sangweni@mrc.ac.za (N.F.S.); pritika.ramharack@mrc.ac.za (P.R.); rabia.johnson@mrc.ac.za (R.J.); 3 Division of Medical Physiology, Faculty of Health Sciences, Stellenbosch University, Tygerberg, Cape Town 7505, South Africa; 4 Applied Microbial and Health Biotechnology Institute, Cape Peninsula University of Technology, Bellville, Cape Town 8001, South Africa; marnewickj@cput.ac.za

**Keywords:** *Protea venus*, phenolic compounds, antioxidant activity, molecular docking, glucolipotoxicity

## Abstract

*Protea venus*, a hybrid of *Protea repens* and *Protea aristata*, is a commonly found flower in the South African market. To date, there are no reported chemical or biological studies on this hybrid. This study presents the first investigation of the chemical composition and preliminary biological activity of the methanolic extract of *P. venus*. Phytochemical analysis of the methanolic extract led to the isolation of nine known phenolic compounds (**2**–**10**) and one new compound, *p*-coumaroyl calleryanin (**1**). The identified known constituents include calleryanin derivatives (**2**–**4**), lacticolorin (**5**), quercetin derivatives (**6**–**8**), protocatechuic acid (**9**), and *p*-hydroxybenzoic acid (**10**). Notably, calleryanin (**2**), protocatechuoyl calleryanin (**3**), kaempferol-3-*O*-rhamnoside (**6**), and quercetin-3-*O*-rhamnoside (**7**) are reported for the first time in the genus *Protea*. Compounds **3** and **9** exhibited strong antioxidant activity in the ferric reducing antioxidant power (FRAP) assay, with 9 exceeding vitamin C. Molecular docking studies suggest that the isolated compounds may interact with Kelch-like ECH-associated protein 1 (KEAP1). In H9c2 cardiomyocytes exposed to high glucose (40 mM) and palmitate (0.15 mM), the extract and compound **6** were non-cytotoxic (≤100 µg/mL) and produced a moderate restoration of ATP levels under glucolipotoxic conditions. These findings expand the phytochemical profile of *P. venus* and provide preliminary insight into its biological activity under metabolic stress.

## 1. Introduction

The genus *Protea* (Proteaceae), commonly known as sugarbushes, comprises approximately 136 species, nearly 90 of which are endemic to South Africa [1,2]. Beyond their economic and ornamental importance [3,4], *Protea* species have attracted increasing attention due to their rich phytochemical diversity and associated biological activities [5,6,7]. Despite this growing interest, the genus remains relatively underexplored, with only a small number of species investigated in detail and fewer than 60 compounds fully characterized to date [5,7].

Natural products, particularly phenolic compounds, have been widely investigated for their ability to modulate oxidative stress and inflammation, processes central to chronic diseases such as diabetes mellitus [8]. Diabetes is a major global health challenge and a leading cause of cardiovascular complications, including diabetic cardiomyopathy (DCM), a condition characterized by cardiomyocyte dysfunction, metabolic disturbances, oxidative stress, and inflammation, independent of coronary artery disease or hypertension [9,10,11,12].

Phytochemical studies indicate that *Protea* species are rich in phenolic compounds, including flavonoids, arbutin derivatives, *bis*-5-alkylresorcinols, and aromatic esters [5,6,7,13]. These metabolites underpin the genus’s phytochemical and pharmacological profile. For example, in *P. cynaroides*, the phenolic acids 3,4-dihydroxybenzoic and 3-hydroxykojic acids exhibit tyrosinase inhibitory properties, whereas caffeic, ferulic, and gallic acids provide antioxidant and anti-inflammatory benefits [6]. Other species yield long-chain fatty alcohols such as 1-heptacosanol (from *P. caffra*), which exhibit antibacterial and antidiabetic activities [14], or phenolic glucosides such as lacticolorin (from *P. lacticolor*), which suppresses histamine release and reduces pro-inflammatory cytokines tumor necrosis factor-alpha (TNF-α) and interleukin-6 (IL-6) in human mast cells [15]. Additionally, cytotoxic isoflavones from *P. gaguedi*, including 5-methoxydurmillone and jamaicin, exhibit anticancer potential against the human carcinoma cell line KB-3-1 [16].

Despite these promising findings, most species of the genus remain chemically unexplored. Considering the therapeutic potential of previously identified phenolic compounds, the genus is an untapped source of bioactive metabolites. Importantly, hybrid species within the genus remain completely uninvestigated, despite their potential to generate novel phytochemical profiles through genetic recombination. A prime example of this research gap is *P. venus*, a hybrid of *P. repens* and *P. aristata*, widely cultivated in the cut-flower industry for its vibrant red inflorescences [17]. While horticulturally valued, its phytochemical profile has not been reported. Therefore, this study aims to investigate the secondary metabolites of *P. venus* and to conduct a preliminary assessment of its biological potential, thereby expanding our understanding of the chemical diversity within this genus and providing a basis for future pharmacological research.

## 2. Results

### 2.1. Purification of Protea venus Chemical Constituents

**Figure 1:**
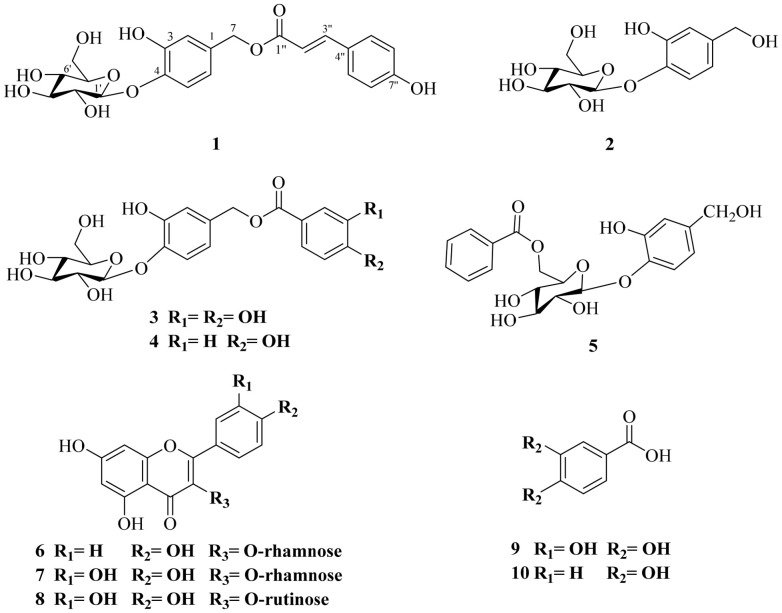
Chemical structures of the isolated compounds (**1**–**10**) from *Protea venus*.

Phytochemical analysis of the methanolic leaf extract of *P. venus* led to the isolation of ten compounds (**1**–**10**). Compound **1** (Figure 1, Table 1) was identified as a calleryanin derivative and isolated for the first time, while compounds **2** (calleryanin), **3** (protocatechuoyl calleryanin) [18], **6** (kaempferol-3-*O*-rhamnoside) [19] and **7** (quercetin-3-*O*-rhamnoside) [20] are reported for the first time from the genus *Protea*. Other compounds included *p*-hydroxybenzoyl calleryanin (**4**), lacticolorin (**5**), rutin (**8**), protocatechuic acid (**9**), and *p*-hydroxybenzoic acid (**10**).

### 2.2. Total Phenolic Content and Antioxidant Activity of the Crude Extract and Isolated Compounds

Crude extract and compounds (**3**–**6** and **9**) with enough quantity (>3.0 mg) were subjected to antioxidant analysis. The crude extract showed a high total phenolic content (199.05 ± 1.64 mg GAE/g). The ferric reducing antioxidant power (FRAP) assay was performed according to Benzie and Strain’s method [18], and antioxidant activity was expressed as μmol equivalents per gram of sample (μmol AAE/g). Compounds **3** and **9** demonstrated strong ferric reducing power, with compound **9** exhibiting greater activity than vitamin C. In contrast, compounds **4**–**6** and the crude extract displayed comparatively weak FRAP activity (Table 2).

The Trolox equivalent antioxidant capacity (TEAC) assay was conducted following the method of Pellegrini et al. [19], and results were expressed as μmol Trolox equivalents per gram of sample (μmol TE/g). Vitamin E served as the positive control and exhibited strong radical scavenging. The crude extract showed weak activity, whereas compounds **3**–**6** and **9** showed moderate scavenging effects.

### 2.3. Molecular Docking

Molecular docking was performed against the KEAP1 (PDB ID: 2FLU) using ML334 and RA839 as reference ligands. ML334 exhibited a docking score of −7.6 kcal/mol, whereas RA839 showed a docking score of −6.0 kcal/mol (Table S2). Among the isolated compounds, compound **5** displayed the highest binding affinity (−7.8 kcal/mol), followed by compounds **6** (−7.6 kcal/mol), **3** (−7.4 kcal/mol), and **4** (−7.0 kcal/mol). Compound **9** exhibited the weakest interaction profile and the lowest binding affinity.

**Figure 2:**
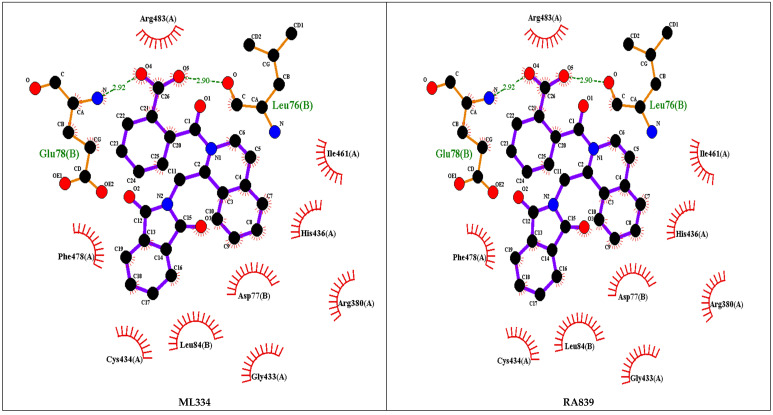

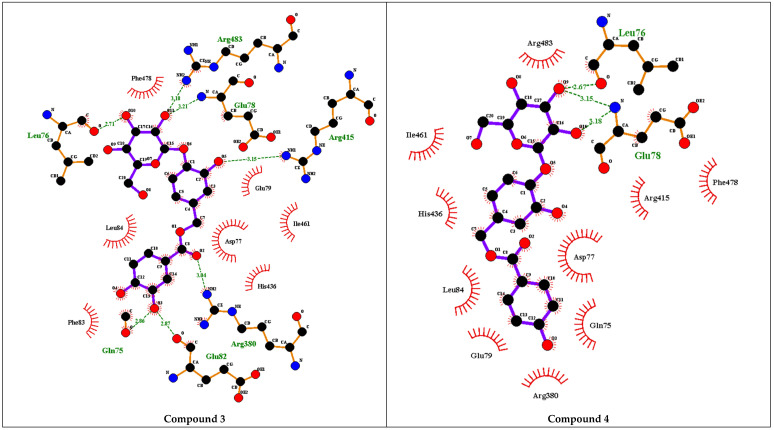

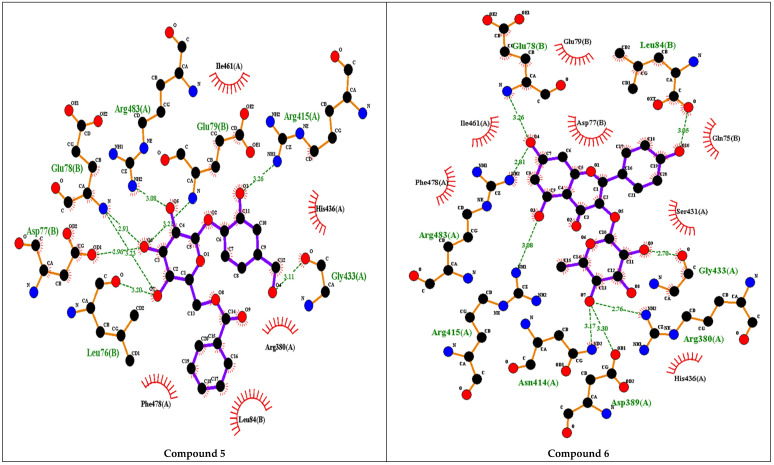

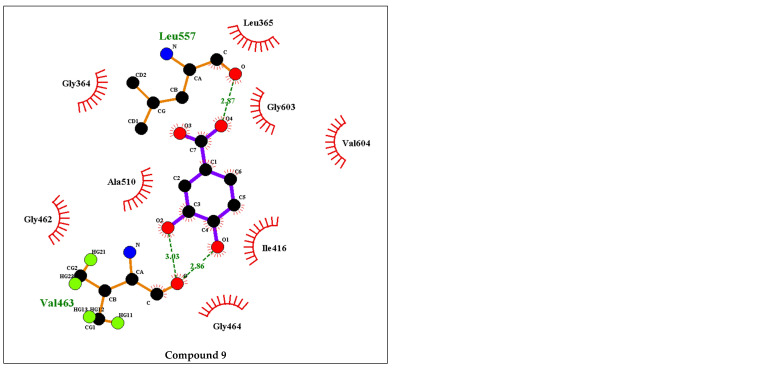
LigPlot 2D schematic representations of the interactions between the docked compounds (**3**–**6** and **9**) and KEAP1. Hydrogen bonds are depicted as dashed lines, whereas hydrophobic contacts are represented by arcs with spokes directed toward the interacting ligand atoms. Protein residues involved in ligand binding are labeled in each diagram.

Ligand interaction analysis (Figure 2, Table S2) showed that both ML334 and RA839 formed hydrogen bonds with Glu78 and Leu76. Compound **4** reproduced this hydrogen-bonding pattern, forming hydrogen bonds with Glu78 and Leu76, and engaging in hydrophobic interactions with Arg380, Arg483, Arg415, Asp77, Gln75, Glu79, His436, Ile461, and Leu84. Compounds **3** and **5** also formed hydrogen bonds with Glu78 and Leu76, as well as with several other residues. Compound **3** formed hydrogen bonds with Arg483, Arg415, Arg380, Glu82, and Gln75, whereas compound **5** formed hydrogen bonds with Arg483, Arg415, Asp77, Glu79, and Gly433. Compound **6** retained a hydrogen bond with Glu78 but did not interact with Leu76. Instead, compound **6** formed hydrogen bonds with Arg483, Arg415, Arg380, Asp389, Leu84, Gly433, and Asn414.

Several residues involved in hydrophobic interactions with the reference ligands formed hydrogen bonds with compounds **3**, **5**, and **6**. In particular, Arg483, Arg415, Arg380, and Gly433 were involved in hydrogen-bonding interactions with these compounds, whereas they contributed primarily to hydrophobic interactions in the reference ligands. Compound **9** displayed a distinct interaction pattern characterized predominantly by hydrophobic contacts with Gly364, Gly462, Gly603, Gly464, Ala510, Ile461, and Val604 and a single hydrogen bond with Leu557.

### 2.4. Cytotoxicity Assessment of Protea venus Crude Extract and Selected Compounds

**Figure 3:**
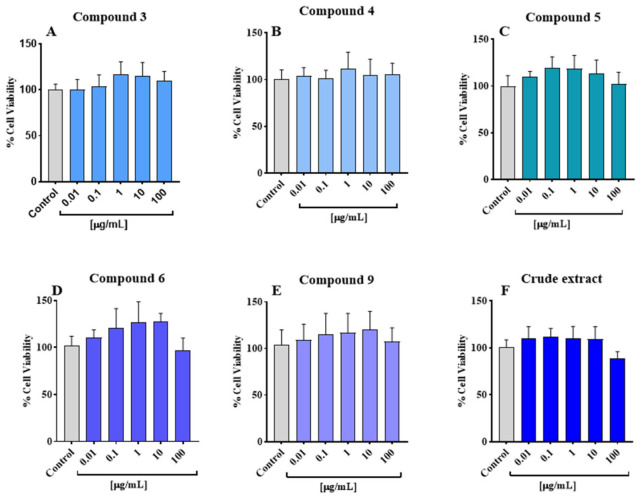
Cell viability assessment using the MTT assay. H9c2 cardiomyoblasts were treated for 24 h with *P. venus* isolated compounds and crude extract. (**A**–**E**) Compounds **3**–**6**, **9**, (**F**) Crude extract. Data presented as mean ± SD, *n* = 3. Control = 0.53% DMSO.

The cytotoxicity of the methanolic extract and compounds **3**–**6** and **9** was assessed in H9c2 cardiomyoblasts using the MTT assay after 24 h of exposure (Figure 3). Comparison of the untreated and vehicle control groups revealed no significant differences in cell viability (Figure S5), demonstrating that the final DMSO concentration (0.53%) did not exert detectable cytotoxic effects. Therefore, all subsequent analyses were normalized to the vehicle control.

### 2.5. Effects of Protea venus Isolated Compounds and Crude Extract on Adenosine Triphosphate (ATP) Production in H9c2 Cells Exposed to High Glucose and Palmitate

Exposure of H9c2 cardiomyocytes to glucolipotoxic conditions (40 mM glucose and 150 µM palmitate) markedly reduced intracellular ATP levels to 50.06 ± 1.31 compared with the normal glucose control group (100.28 ± 5.07). Statistical comparisons were conducted against the HG + Pal group. Accordingly, the normal glucose control exhibited significantly higher ATP levels (*p* < 0.0001), confirming the detrimental effects of glucolipotoxic stress on cellular energy metabolism.

**Figure 4:**
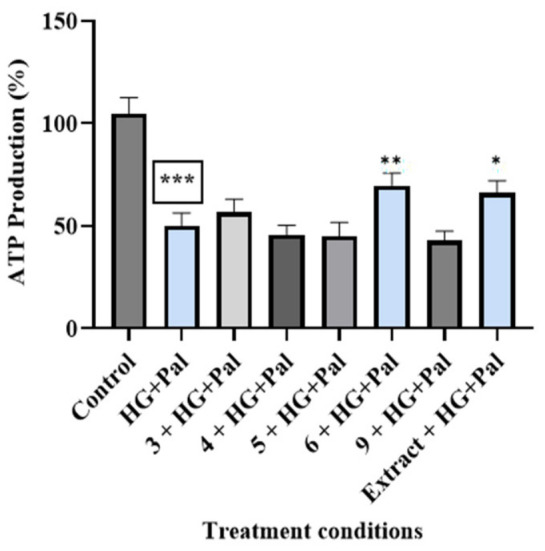
Effect of the methanolic extract of *Protea venus* and isolated compounds (**3**–**6** and **9**) on intracellular ATP levels in H9c2 cardiomyocytes under glucolipotoxic conditions. Cells were exposed to high glucose (40 mM) and palmitate (150 µM) for 24 h, followed by treatment with the extract or compounds (10 µg/mL) for an additional 24 h. Intracellular ATP levels were quantified using a luminescence-based assay and normalized to total protein content. Data were normalized to the vehicle control and are presented as mean ± SEM from 3 independent biological experiments, each performed with 4 technical replicates (*n* = 3). Statistical significance was determined by one-way ANOVA followed by Dunnett’s multiple-comparison test, with all groups compared against the glucolipotoxic (HG + Pal) group. * *p* < 0.05, ** *p* < 0.01, and *** *p* < 0.001.

Treatment with the methanolic extract partially restored ATP levels to 60.90 ± 1.37 (*p* < 0.05), while compound **6** increased ATP levels to 61.40 ± 1.47 (*p* < 0.01). In contrast, compounds **3**, **4**, **5**, and **9** did not significantly affect ATP levels (Figure 4).

These findings indicate that the methanolic extract and compound **6** partially attenuate glucolipotoxicity-induced ATP depletion.

## 3. Discussion

### 3.1. Structure Elucidation of p-Coumaroyl Calleryanin (**1**)

Compound **1**, a white amorphous powder, was assigned to the molecular formula C_22_H_24_O_10_ by HR-ESIMS (negative mode, Figure S1), which showed a deprotonated molecule [M-H]^−^ at *m*/*z* 447.1285 (calculated for C_22_H_23_O_10_, 447.1297). The UV absorbance in methanol showed three major peaks typical for benzene ring and coumarate ester moiety at wavelengths (λ_max_) 222, 254, and 264 nm (Figure S2). The FTIR spectrum (Figure S3) exhibits characteristic bands at 3370 cm^−1^ (O–H stretch) and 2922 cm^−1^ (aliphatic C–H stretch), along with a conjugated ester carbonyl (C=O) stretch at 1688 cm^−1^. Aromatic ring skeletal (C=C) stretching is confirmed by signals at 1612, 1604, and 1513 cm^−1^, supplemented by benzylic and glucosyl C–H bending at 1438 and 1379 cm^−1^. The ester linkage and phenol hydroxyl groups are marked by C–O stretches at 1281, 1180, and 1169 cm^−1^, while the glucopyranoside core is validated by the aliphatic C–O stretch at 1034 cm^−1^.

The ^1^H NMR spectrum showed seven distinctive aromatic proton peaks at δ_H_ 6.89 (*d*, *J* = 1.7 Hz; H-2), 7.19 (*d*, *J* = 8.1 Hz, H-5), 6.83 (*dd*, *J* = 8.1, 1.7 Hz, H-6), 7.46 (*d*, *J* = 8.2 Hz, H-5″ and H-9″), and 6.79 (*d*, *J* = 8.6 Hz, H-6″ and H-8″). Moreover, two olefinic proton peaks at δ_H_ 6.34 (*d*, *J* = 15.7 Hz, H-2″) and 7.62 (*d*, *J* = 15.7 Hz, H-3″) with vicinal coupling constants of 15.7 Hz, typical of a *trans*-oriented double bond, were detected. A methylene unit at δ_H_ 5.10 (*s*, H-7) was observed in the downfield region. Additionally, an anomeric proton at δ_H_ 5.16 (*d*, *J* = 8.4 Hz, H-1′) and a cluster of hydroxymethine signals in the sugar region from δ_H_ 3.59 to 4.15, characteristics of glucopyranosyl moiety, were observed.

The ^13^C NMR spectrum displayed 22 distinctive signals including a carbonyl group at δ_C_ 167.6 (C-1″); fourteen methine groups at δ_C_ 115.7 (C-2), 117.1 (C-5), 119.6 (C-6), 113.7 (C-2″), 145.4 (C-3″), 129.8 (C-5″ and C-9″), 115.4 (C-6″ and C-8″), 100.6 (C-1′), 74.5 (C-2′), 71.4 (C-5′), 67.1 (C-3′), and 70.8 (C-4′); two methylene groups at δ_C_ 65.5 (C-7), and 61.3 (C-6′), and five quaternary carbons at δ_C_ 131.7 (C-1), 147.0 (C-3), 145.5 (C-4), 125.8 (C-4″), and 159.9 (C-7″).

**Figure 5:**
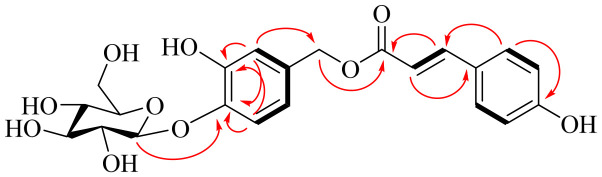
Key COSY (**—**) and HMBC (→) correlations of compound **1**.

The HMBC spectrum showed that the anomeric proton H-1′ correlates with C-4, indicating that the glucopyranosyl moiety is attached to ring A at position 4. The ^1^H-^1^H COSY spectrum showed correlations among H-2, H-5, and H-6, confirming that ring A is a trisubstituted benzene ring (Figure 5). Furthermore, the HMBC spectrum showed that the aromatic protons H-2 with C-1; C-7; C-3; and C-4 and H-6/C-1 and C-4, identifying the aglycone as 3,4-dihydroxybenzyl alcohol, which is linked to a glucose in the 4-position, commonly known as calleryanin, a common metabolite from *Protea* species [20].

The chemical shifts of C-5″, C-6″, C-8″, and C-9″ suggested the presence of a 1,4-disubstituted benzene ring. This was further supported by HMBC correlations between H-5″ and H-9″/C-3″, C-7″, C-5″, and C-9″. Peaks associated with the *p*-hydroxybenzene ring also showed HMBC correlations of H-2″, H-3″ with C-5″, C-1″, confirming the presence of a double bond attached to the benzene ring, thereby confirming the presence of a *p*-coumaroyl moiety. This was further supported by the MS fragment at 163.0390, which corresponds to the coumarate fragment (Figure S1). The oxymethylene proton (H-7) correlated with C-1″ (HMBC), indicating that the *p*-coumaroyl unit is attached to C-7. The observed optical rotation value of ${\left[a\right]}_{D}^{25}$: −13.3 is consistent with previously reported data for related calleryanin ester derivatives [21]. Additionally, the downfield shift of CH_2_-7 in compound **1** (δ_H_ 5.10, s) compared to **2** (δ_H_ 4.48, s) confirms esterification at C-7. This value is in close agreement with those of the analogous ester derivatives, compound **3** (δ_H_ 5.13) and compound **4** (δ_H_ 5.16). All other proton signals for compound **1** were similar to those of **2**. Together, these findings confirm that compound **1** is a derivative of calleryanin and is identified as *p*-coumaroyl calleryanin.

**Figure 6:**
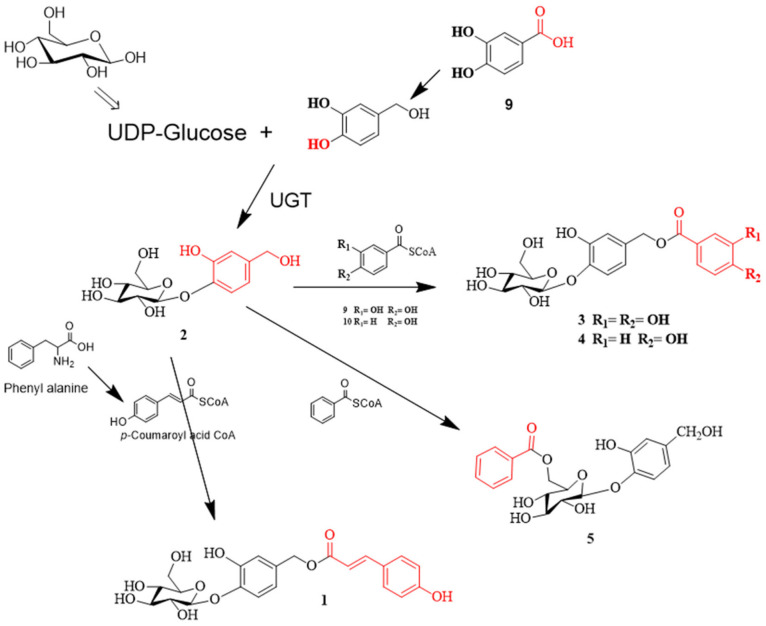
Hypothetical biosynthetic scheme illustrating the biogenetic correlation among the isolated phenolic derivatives.

In addition to compound **1**, four known calleryanin derivatives (**2**–**5**) were isolated, providing insight into the *P. venus* chemical composition and suggesting a common biosynthetic origin. Calleryanin (**2**) has previously been reported alongside its protocatechuoyl and hydroxybenzoyl esters in *Pyrus* species [20], indicating that these metabolites commonly occur as structurally related phenolic glycosides. The occurrence of acylated derivatives differing in the position and nature of the acyl substituent is consistent with diversification through acyltransferase-mediated modification of a common precursor during plant secondary metabolism [22]. The structural coexistence of compounds **1**–**5**, **9**, and **10** can be biogenetically correlated via the scheme illustrated in Figure 6. The pathway initiates with the formation of glucoside intermediate **2**. This intermediate undergoes regioselective esterification at the glucose ring with various benzoyl-CoA derivatives to produce compounds **3**–**5** (including derivatives **9** and **10**). Meanwhile, acylation directed at the primary aliphatic hydroxyl group of the benzyl alcohol unit of **2** yields compound **1**.

Calleryanin (**2**), previously identified in *P. lacticolor* [23], *Pyrus calleryana* [20], and *Astragalus membranaceus* [24], has demonstrated in vivo anti-inflammatory effects greater than those of acetylsalicylic acid [25]. Compound **3** is identified as protocatechuoyl calleryanin, also known as oreganol A [26], oblongaroside C [27] and odontoside [28]. It has been detected in oregano [26] and *Pyrus calleryana* [20], and has demonstrated several biological activities such as anti-neurotropic, nephrotropic (diuretic) [26] and anti-tyrosinase [29] activities. Similarly, *p*-hydroxybenzoyl calleryanin (**4**), isolated from *P. calleryana* [20] and *P. cynaroides* [30], showed antifungal activity [30].

In addition to these glycosides, flavonoids such as quercetin and its glycoside, rutin (**8**), have been widely reported to exert cytoprotective effects by attenuating oxidative stress, modulating inflammatory signaling, and reducing apoptosis in both cardiac and non-cardiac cells, including H9c2 cardiomyocytes [31,32,33,34].

Protocatechuic acid (**9**) has likewise demonstrated protective effects in models of ischemia–reperfusion injury, adrenergic stress-induced cardiac hypertrophy, and doxorubicin-associated cardiotoxicity, consistent with its antioxidant and anti-inflammatory properties [35,36].

The presence of these compounds in *P. venus* is consistent with known structure-activity relationships for polyphenolic metabolites, where phenolic hydroxylation and acyl substitution patterns may influence their reported antioxidant, cytoprotective, and anti-inflammatory properties [37,38,39,40].

### 3.2. Antioxidant, In Silico and In Vitro Assessment of Selected Protea venus Phenolics Under Metabolic Stress

The stronger FRAP activity of compounds **3** and **9** is likely attributable to the presence of a catechol (3,4-dihydroxyphenyl) moiety, which facilitates electron donation and stabilizes the resulting phenoxyl radical through both resonance delocalization and intramolecular hydrogen bonding between adjacent hydroxyl groups [37].

In contrast, compound **4** bears only a single hydroxyl group on the aromatic ring, limiting its reducing capacity. The weaker activity of compounds **5** and **6** may similarly reflect the absence of a catechol unit and, in the case of compound **6**, glycosylation, which can reduce the availability of free phenolic groups for electron transfer [38]. Collectively, these findings suggest that the number and arrangement of phenolic hydroxyl groups are key determinants of ferric reducing power among the isolated compounds.

Docking against the KEAP1 identified several compounds with binding affinities comparable to or greater than those of the reference ligands ML334 and RA889. These reference ligands provide a useful framework for evaluating the binding characteristics of the isolated compounds within the KEAP1 Kelch domain [41,42]. Interestingly, compound **4** reproduced the hydrogen-bonding pattern observed for both reference ligands through interactions with Glu78 and Leu76, yet did not exhibit the strongest binding affinity. This observation suggests that conservation of key residue interactions alone may not fully account for ligand binding within the KEAP1 pocket.

In contrast, compounds **3**, **5**, and **6** exhibited distinct interaction profiles in which residues that participated predominantly in hydrophobic interactions with reference ligands formed hydrogen bonds with the test compounds. These findings indicate that the isolated compounds can achieve favorable binding affinities through interaction patterns that differ from those of the reference ligands. Among these compounds, compound **5** exhibited the strongest predicted binding affinity toward KEAP1, whereas compound **6** displayed a binding affinity equivalent to ML334.

Despite their favorable docking scores, compounds **3**–**5** did not improve intracellular ATP levels under glucolipotoxic conditions. Only compound **6** significantly attenuated ATP depletion among the isolated compounds, while the methanolic extract also produced a significant increase in ATP levels. This discrepancy underscores the importance of integrating computational predictions with functional validation when evaluating potential therapeutic candidates and suggests that predicted binding affinity alone may not reliably predict biological activity.

Interestingly, compounds **3** and **9** exhibited the strongest antioxidant activities in the FRAP assay but did not significantly improve ATP levels under glucolipotoxic conditions. Conversely, compound **6** significantly attenuated ATP depletion despite exhibiting comparatively weaker FRAP activity. These findings indicate that neither a strong ferric-reducing capacity nor favorable KEAP1-binding predictions necessarily translates into improved cellular bioenergetics. Rather, preservation of intracellular ATP levels appears to depend on factors beyond those captured by cell-free antioxidant assays and molecular docking simulations.

The ATP-restorative effect of the crude extract was comparable to that of compound **6**, despite the extract comprising a complex mixture of constituents. This observation suggests that the extract’s biological activity may not be attributable to a single constituent. Given the chemical complexity of plant extracts, multiple phytochemicals may collectively contribute to the observed activity through additive or synergistic interactions. Such effects have been reported for phenolic-rich plant extracts, where structurally diverse constituents may act through complementary mechanisms to influence cellular responses [8]. Nevertheless, dedicated combination studies, such as fixed-ratio dose–response assays, would be required to determine whether such effects reflect synergism, simple additivity, or the activity of individual constituents.

## 4. Materials and Methods

### 4.1. Chemicals, Reagents, and Equipment

The Silica gel 60 (0.063–0.200 mm), Sephadex (LH-20), and aluminium thin layer chromatography plate supplied by Merck (Cape Town, South Africa) were used. Hexane, ethyl acetate, and methanol AR grades were purchased from the local market (Kimix, Cape Town, South Africa). The Shimadzu LC-20 HPLC system (Shimadzu Corporation, Kyoto, Japan) has a quaternary pump (LC-20AD), a diode array detector (DAD, SPD-M20A), and a manual injector. HPLC-grade methanol and a reversed-phase C18 column (25 × 1 cm, 5 μm, Supelco, Merck, South Africa) were used for purification. The detector was operating at wavelengths 254, 272, and 366 nm. The 1D NMR (^1^H, ^13^C, and DEPT-135) and 2D NMR (HMBC, HSQC, and CO-SY) spectra were measured using a Bruker spectrometer (Rheinstetten, Germany) operating at 400/100 MHz for ^1^H and ^13^C NMR, respectively.

### 4.2. Isolation of Chemical Constituents

*P. venus* was collected from Kirstenbosch National Botanical Garden (KBG), South Africa, in August 2021 under permit No. CN35-28-9937, dated 23 July 2019, the herbarium specimen was identified by the KBG team and kept in the Chemistry Department.

The fresh leaves (~1.3 kg) were blended with methanol and left at room temperature for 24 h. After filtration, the combined filtrates were concentrated to yield a dark-green crude extract (70 g, 5.3% fresh weight). The extract (70.0 g) was fractionated over silica gel 60 using stepwise gradients of hexane/ethyl acetate followed by ethyl acetate/methanol, yielding 8 major fractions (I–VIII). Fractions with similar TLC profiles were combined based on Rf values. Fractions I–IV were low in quantity (less than 30 mg) and were excluded from further purification.

Fraction VI (12.59 g), upon standing, formed crystalline precipitates that were separated from the corresponding supernatant and identified as compound **5** (2.18 g). The supernatant fraction (8.66 g) was subjected to isocratic column chromatography using ethyl acetate-methanol (95:5, *v*/*v*), followed by semi-preparative HPLC on the resulting fractions. All semi-preparative HPLC was performed using HPLC-grade methanol and deionized water at a flow rate of 1.0 mL/min, with a gradient from 60% to 80% methanol over 45 min, followed by a gradient from 80% to 100% methanol over 15 min. This yielded compounds **2** (2.1 mg, R_t_ 18.2 min), **9** (4.8 mg, R_t_ 22.0 min), **10** (2.2 mg, R_t_ 24.4 min), **1** (3.3 mg, R_t_ 30.0 min), **4** (36.3 mg, R_t_ 31.5 min), **3** (24.4 mg, R_t_ 38.5 min), **6** (9.5 mg, R_t_ 40.5 min), and **7** (2.1 mg, R_t_ 41.5 min). Fraction VII (20.80 g) yielded a white crystalline precipitate, which was identified as glucose (5.85 g). Fraction VIII (100 mg) was purified by Sephadex LH-20 column chromatography using 80% aqueous methanol to yield compound **8** (2.7 mg).

Compound **1**: (3.3 mg, Rt; 30 min); amorphous white-yellowish powder. UV λ_max_ 222, 254, and 264 nm (MeOH). FTIR: 3370, 2922, 1688, 1612, 1604, 1513, 1438, 1379, 1281, 1180, 1169, and 1034 cm^−1^. ${\left[a\right]}_{D}^{25}$: −13.3 (c: 0.02; MeOH); ^1^H- and ^13^C-NMR see Table 1. HRESIMS *m*/*z* 447.1285 [M-H]^−^ (calcd for C_22_H_23_O_10_, 447.1297).

### 4.3. Biological Assay

Isolated compounds (**1**, **2**, **7** and **10**) with masses below 3 mg were not subjected to cell-free and cell-based assays because the quantities obtained after purification were insufficient for biological evaluation.

#### 4.3.1. Antioxidant Activity

Antioxidant capacity was evaluated using the FRAP (Ferric Reducing Antioxidant Power) and TEAC (Trolox Equivalent Antioxidant Capacity) assays. FRAP was performed according to Benzie and Strain [18], and TEAC was carried out following Pellegrini et al. [19].

#### 4.3.2. Cell Culture

H9c2 rat cardiomyoblasts were cultured in Dulbecco’s Modified Eagle’s Medium (DMEM) supplemented with 10% fetal bovine serum (FBS) under standard conditions (37 °C, 5% CO_2_, humidified atmosphere).

#### 4.3.3. Cytotoxicity

Cells were seeded in clear 96-well plates at a density of 0.8 × 10^5^ cells/well and treated for 24 h with isolated compounds (0.01, 0.1, 1, 10, and 100 µg/mL) prepared in complete culture medium.

#### 4.3.4. Medium Preparation

A 100 mM palmitate stock solution was prepared by dissolving 0.256 g of palmitic acid in 10 mL of absolute ethanol and heating at 70 °C until completely dissolved [43]. To generate glucolipotoxic conditions, a high-glucose medium was prepared by supplementing 25 mM DMEM (4.5 g/L glucose) with D-glucose to reach a final concentration of 40 mM [44,45]. To control for osmotic effects, 15 mM mannitol was added to the normal glucose control medium to match the osmolarity of the high-glucose treatment [46].

#### 4.3.5. Palmitate Preparation

For palmitate conjugation, fatty-acid-free bovine serum albumin (BSA) was dissolved in DMEM to a final concentration of 2% (*w*/*v*; approximately 300 µM). Sodium palmitate stock was subsequently added to achieve a final working concentration of 150 µM (representing a 2:1 BSA-to-palmitate molar ratio). The solution was incubated at 37 °C for at least 3 h until completely clear. A corresponding BSA-only vehicle control (25 mM) was included and used as control. The glucolipotoxic medium was then filter sterilized prior to use [47].

#### 4.3.6. Glucolipotoxicity Induction and Assessment of Intracellular ATP Level

Glucolipotoxicity was induced by exposing cells to high glucose (40 mM) and palmitate (150 µM) for 24 h.

Based on the results of the MTT cytotoxicity assay, a concentration of 10 µg/mL was selected for the methanolic extract and isolated compounds for subsequent ATP experiments. At this concentration, none of the test samples significantly reduced H9c2 cell viability, indicating they were sub-cytotoxic. Following glucolipotoxic induction, cells were treated with the methanolic extract or isolated compounds for a further 24 h.

Intracellular ATP levels were quantified using a luminescence-based ATP assay according to the manufacturer’s instructions and normalized to total protein content. Data are expressed as mean ± SEM from three independent biological experiments, each performed with five technical replicates per treatment condition.

#### 4.3.7. Statistical Analysis

##### TEAC and FRAP Assay

Values are expressed as mean ± SD (*n* = 3). Different superscript letters indicate significant differences according to two-way ANOVA followed by Tukey’s multiple comparisons test (*p* < 0.05).

##### ATP Assay

Experiments were evaluated using five technical replicates across three independent biological experiments (*n* = 3). Statistical analyses were performed in GraphPad Prism 9.5. Data are presented as mean ± SEM. Differences between groups were analyzed using one-way ANOVA with Dunnett’s multiple-comparisons test, and *p* < 0.05 was considered statistically significant.

#### 4.3.8. Phytochemical Compounds Acquisition and Geometry Optimization

The 3D structures of the tested compounds were obtained from the PubChem database and geometrically optimized using Avogadro (version 1.1.0) through energy minimization. Avogadro is integrated with the GHEmical and UFF force fields to optimize the molecular geometries of natural compounds, using the steepest-descent algorithm for structural minimization [48]. All the compounds were generated as .mol2 files, which are recognized as ligand input in UCSF Chimera.

#### 4.3.9. Protein Acquisition, Preparation, and Molecular Docking

The protein crystal structure of KEAP1 (PDB ID: 2FLU) [49] was obtained from the RSCB PDB as .pdb files, which are recognized as receptor input files in UCFS Chimera [50]. Before docking, the proteins underwent thorough preparation, including removal of nonessential substrates such as water. The protein structure was further prepared for molecular docking using the dock prep module in UCSF Chimera (version 1.11.2) to add hydrogens, perform partial residue replacement using Dunbrack rotamer library, convert bromo-UMP to UMP, convert selenomethionine to methionine, and convert methylsulfonyl-dCMP to CMP. The AMBER.ff14SB force field was employed to distribute the Gasteiger charges on the protein molecule. Then Docking was conducted using grid boxes encompassing the active-site residues of KEAP1 (Table S1).

## 5. Conclusions

The isolation of 10 compounds, including a new metabolite, highlights *Protea venus* as a chemically diverse source of phenolic constituents. Docking, antioxidant, and ATP assays revealed distinct activity profiles among the isolated compounds. Although compound **5** exhibited the strongest predicted affinity toward KEAP1 and compounds **3** and **9** displayed the highest antioxidant capacities, only compound **6** significantly restored intracellular ATP levels under glucolipotoxic conditions. These findings demonstrate that neither predicted KEAP1 binding affinity nor cell-free antioxidant activity alone is sufficient to predict cellular responses under glucolipotoxic stress, underscoring the importance of integrating computational, chemical, and biological approaches when evaluating potential bioactive compounds.

Overall, this study provides the first phytochemical characterization of *P. venus* and a preliminary evaluation of the biological activities of its constituents in a glucolipotoxic H9c2 cardiomyocyte model. While compound **6** demonstrated the most pronounced ATP-restorative effect under glucolipotoxic conditions, the molecular mechanisms underlying this activity remain unclear. Further studies are required to determine whether these effects are associated with modulation of the KEAP1 signaling pathway or other mechanisms involved in cellular energy homeostasis and stress responses.

## Figures and Tables

**Table 1:** ^1^H and ^13^C NMR data of compounds **1**–**5** (CD_3_OD).

	1		2		3		4		5	
No	δ_C_	δ_H_, *multi.* (*J* = Hz)	δ_C_	δ_H_, *multi.* (*J* = Hz)	δ_C_	δ_H_, *multi.* (*J* = Hz)	δ_C_	δ_H_, *multi.* (*J* = Hz)	δ_C_	δ_H_, *multi.* (*J* = Hz)
1	131.7		137.1		131.3		131.3		137.7	
2	115.7	6.89, *d* (1.7)	114.6	6.86, *brs*	116.2	6.88, *brs*	116.2	6.89, *brs*	117.6	6.76, *brs*
3	147.0		146.9		147.1		147.2		146.9	
4	145.5		144.9		145.8		145.9		144.5	
5	117.1	7.19, *d* (8.1)	117.1	7.17, *d* (8.0)	116.8	7.11, *d* (8.0)	117.1	7.11, *d* (8.4)	116.3	7.02, *d* (8.2)
6	119.6	6.83, *dd* (8.1, 1.7)	118.1	6.78, *brd* (8.0)	119.7	6.82, *brd* (8.0)	119.7	6.81, *brd* (8.4)	114.7	6.51, *brd* (8.2)
7	65.5	5,10, *s*	63.4	4.48, *s*	65.9	5.13, *s*	65.9	5.16, *s*	63.00	4.33, *s*
1′	100.6	5.16, *d* (8.4)	100.9	5.11, *d* (8.0)	100.5	5.00, *d* (7.9)	100.6	4.99, *d* (8.0)	100.6	5.02, *d* (7.9)
2′	74.5	3.80 *	74.6	3.80 *	75.4	3.60 *	75.4	3.67 *	72.2	4.07, *t* (8.2)
3′	67.1	3.59, *dd* (9.0, 2.3)	67.0	3.61 *	67.5	3.41, *d* (9.5)	67.5	3.45 * *m*	68.1	3.57 *
4′	70.8	3.61, *dd* (8.9, 2.3)	70.7	3.63 *	70.8	3.49, *d* (6.3)	70.8	3.45 *	70.7	3.57 *
5′	71.4	4.15, *brs*	71.5	4.19, *brs*	71.5	3.97, *brs*	71.5	3.95, *brs*	71.5	4.02, *brs*
6′	61.3	3.85, *d* (12.0) 3.68, *dd* (12.0, 5.4)	61.3	3.86, *d* (11.4) 3.70 *dd* (11.4, 1.4)	61.5	3.68, *t* (9.5) 3.47 *	61.5	3.67 * 3.45 *	66.2	4.61, *d* (11.4)
1″	167.6				166.0		165.9		166.1	
2″	113.7	6.34, *d* (15.7)			121.0		120.6		130.2	
3″	145.4	7.62, *d* (15.7)			116.7	7.37, *brs*	132.0	7.82, *d* (8.4)	129.7	8.02, *d* (7.5)
4″	125.8				145.5		115.9	6.85, *d* (10.1)	129.2	7.57, *t* (7.9)
5″	129.8	7.46, *d* (8.2)			151.0		162.7		133.9	7.69, *t* (7.9)
6″	115.4	6.79, *d* (8.6)			115.9	6.82, *d* (8.3)	115.9	6.85, *d* (10.1)	129.2	7.57, *t* (7.9)
7″	159.9				122.4	7.34, *brd* (8.3)	132.0	7.82, *d* (8.4)	129.7	8.02, *d* (7.5)
8″	115.4	6.79 *d* (8.6)								
9″	129.8	7.46, *d* (8.2)								

* Partially or fully overlapped signals. *multi.*: multiplicity; *s*: singlet; *brs*: broad singlet; *brd*: broad doublet; *d*: doublet; *dd*: doublet of doublets; *t*: triplet; *m*: multiple.

**Table 2:** Antioxidant activities of different samples from *P. venus*.

Samples	FRAP (µmol AAE/g)	TEAC (µmol TE/g)
Crude extract	1720.57 ± 93.43 ^a^	1207.19 ± 7.31 ^a^
Compound **3**	4192.87 ± 150.22 ^a^	2420.92 ± 0.64 ^a^
Compound **4**	1420.77 ± 47.39 ^a^	2165.57 ± 12.61 ^a^
Compound **5**	1602.96 ± 49.21 ^a^	2380.98 ± 3.15 ^a^
Compound **6**	2228.87 ± 196.03 ^a^	2418.98 ± 5.75 ^a^
Compound **9**	6175.19 ± 63.85	2418.89 ± 0.81
Controls	5743.56 ± 14.43 (vitamin C)	4010.17 ± 41.86 (vitamin E)

The significant difference for the total extract and compounds **3**–**6** and **9** vs. controls was denoted ^a^ for *p* ≤ 0.0001.

## Data Availability

Raw data and data supporting the results can be requested from https://www.cput.ac.za/lib, accessed on 29 June 2026, upon request.

## Supplementary Materials

The following supporting information can be downloaded at: https://www.mdpi.com/article/10.3390/plants15132072/s1, Figure S1: HRESIMS (negative mode) of compound **1**; Figure S2: UV absorbance chromatogram of compound **1** in methanol; Figure S3: IR chromatogram of compound **1**; Figure S4: (a) ^1^H NMR spectrum of compound **1**; (b) ^13^C NMR spectrum of compound **1**; (c) DEPT-135 NMR spectrum of compound **1**; (d) COSY spectrum of compound **1**; (e) HMBC spectrum of compound **1**; (f) HSQC spectrum of compound **1**; Figure S5: Comparative analysis: normal control vs vehicle control; Table S1: Active sites and Gridbox coordinates of the molecular docking simulations; Table S2: Ligand Interaction Plot Analysis of *P. venus* Compounds Bound to KEAP1.
