# Supplementary material for: Isolation of Secondary Metabolites from Protea venus and Evaluation of Their Antioxidant Activity and Effects Under Glucolipotoxic Stress: In Silico and In Vitro Studies
Source: Plants (Basel). 2026 Jul 3;15(13):2072. doi: 10.3390/plants15132072 (PMC13363887; doi:10.3390/plants15132072)
Supplement: Supplementary file 1 [file plants-15-02072-s001.zip › plants-4325794-supplementary.pdf]

Isolation of secondary metabolites from *Protea venus* and evaluation of their antioxidant activity and effects under glucolipotoxic stress: *in silico* and *in vitro* studies

Kadidiatou O. Ndjoubi [1,†], Nonhlakanipho Sangweni [2], Pritika Ramharack [2], Rabia Johnson [2, 3,] Jeanine Marnewick [4] and Ahmed A. Hussein [1,*]

Supplementary data

Figure S1. HRESIMS (negative mode) of compound **1**.

Figure S2. UV absorbance chromatogram of compound **1** in methanol.

Figure S3. IR chromatogram of compound **1**.

Figure S4.a: ^{1}H NMR spectrum of compound **1**.

Figure S4.b: ^{13}C NMR spectrum of compound **1**.

Figure S4.c: DEPT-135 NMR spectrum of compound **1**.

Figure S4.d: COSY spectrum of compound **1**.

Figure S4.e: HMBC spectrum of compound **1**.

Figure S4.f: HSQC spectrum of compound **1**

Figure S5: Comparative analysis: normal control vs vehicle control

Table S1: Active sites and Gridbox coordinates of the molecular docking simulations

Table S2: Ligand Interaction Plot Analysis of P. Venus Compounds Bound to KEAP1

Supplementary data

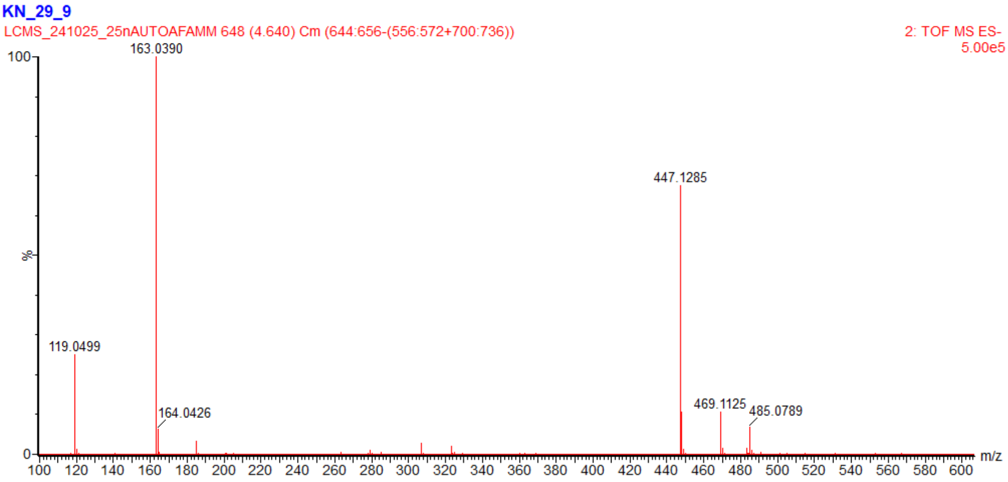

Figure S1. HRESIMS (negative mode) of compound 1.

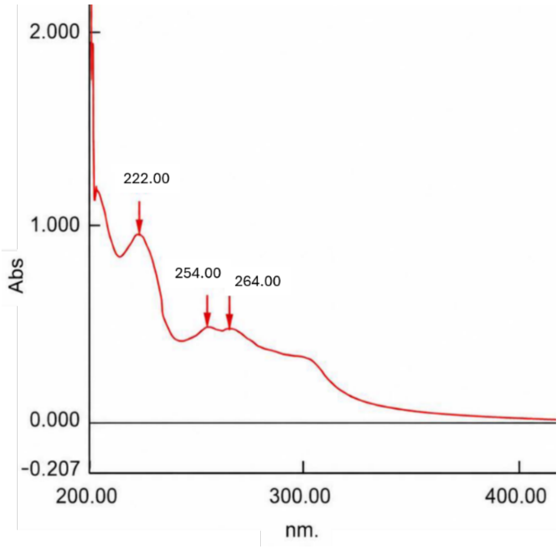

Figure S2. UV absorbance chromatogram of compound 1 in methanol.

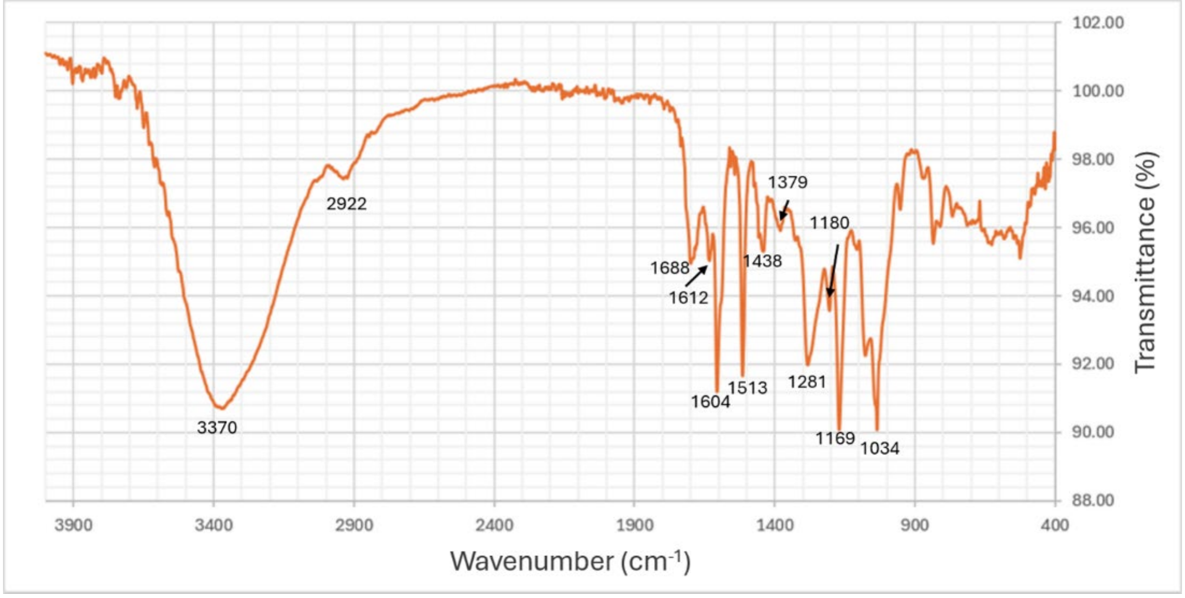

Figure S3. IR chromatogram of compound 1.

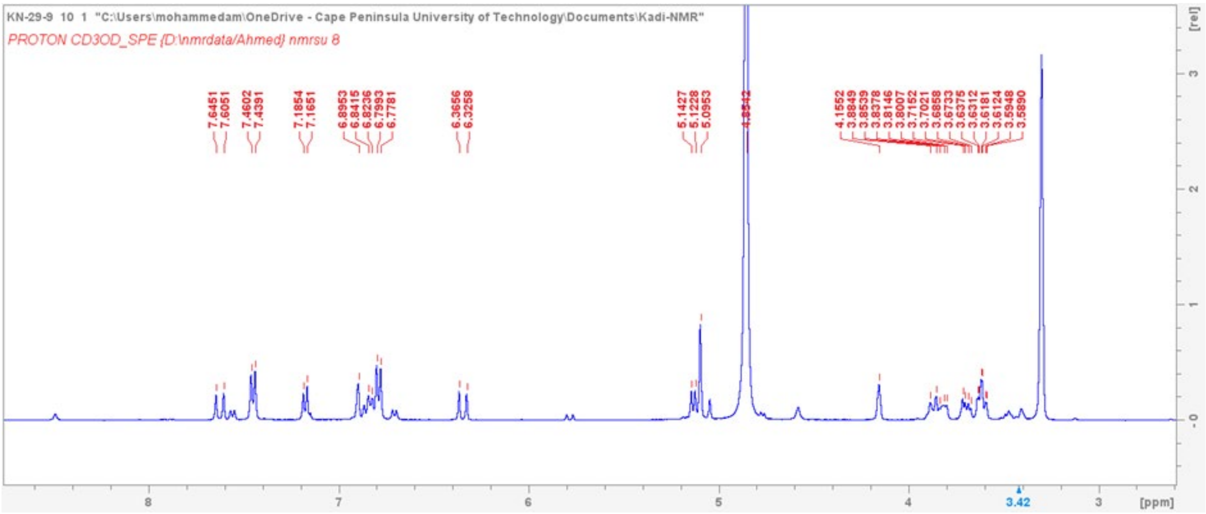

Figure S4.a: ^{1}H NMR spectrum of compound **1.**

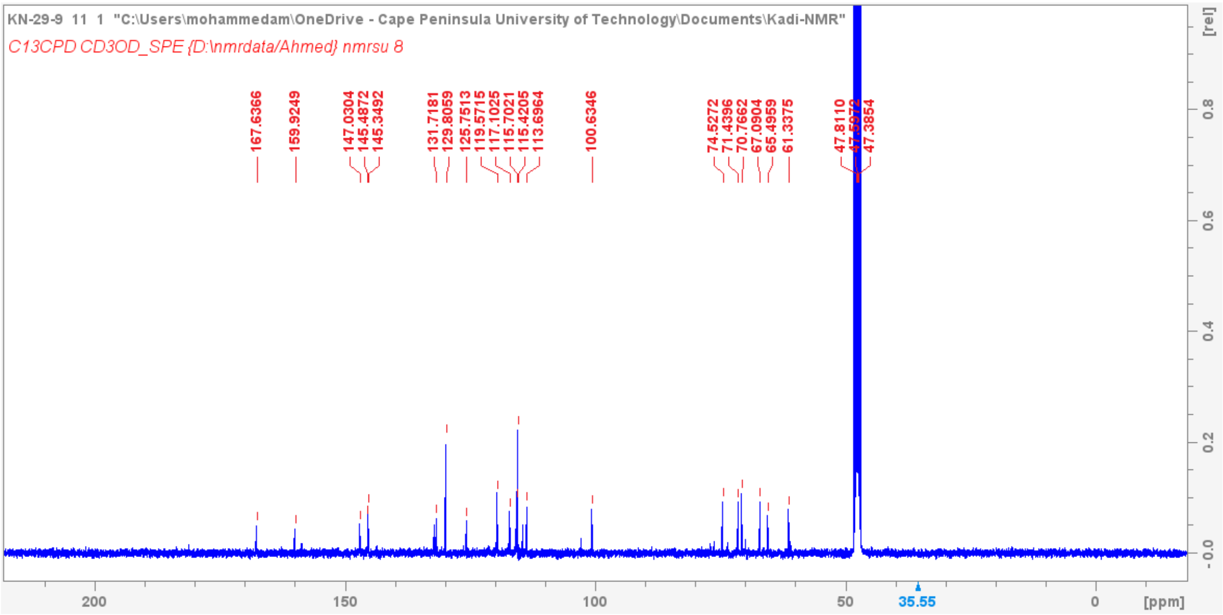

Figure S4.b: ^{13}C NMR spectrum of compound **1**

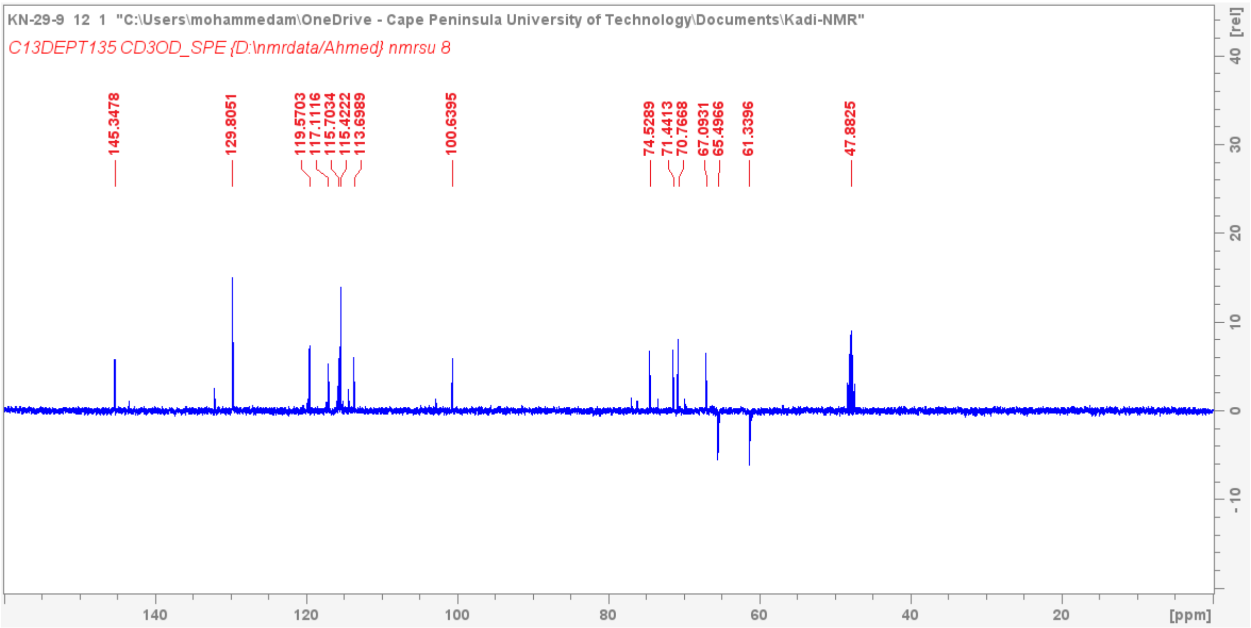

Figure S4.c: DEPT-135 NMR spectrum of compound **1**

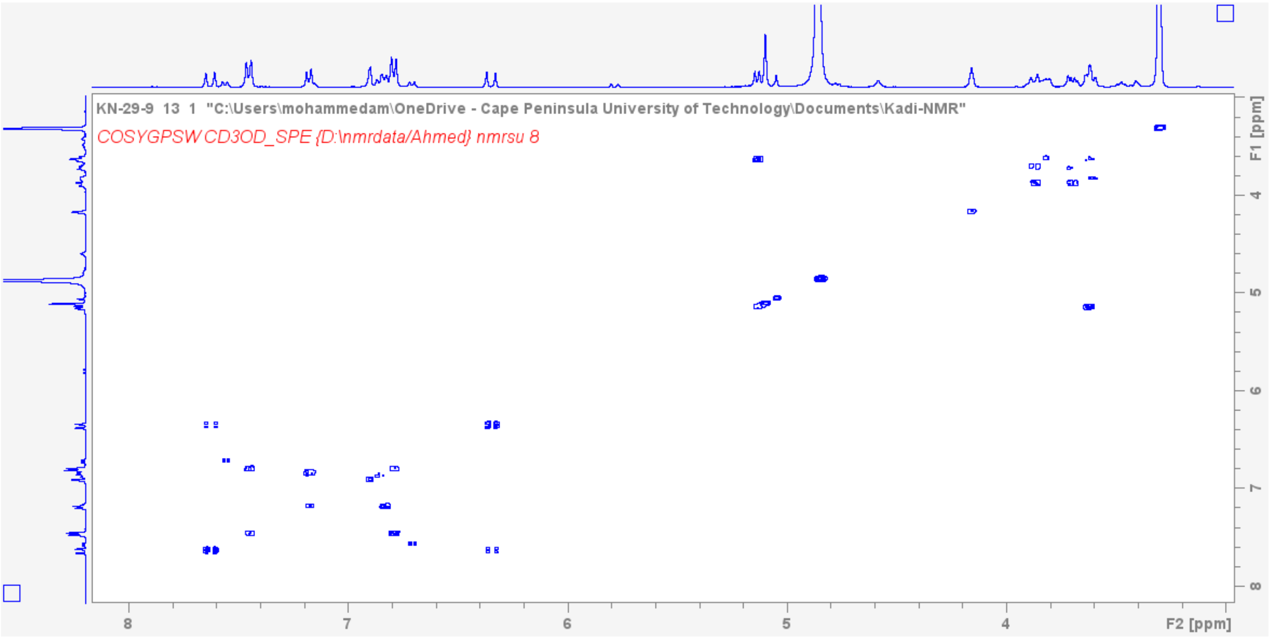

Figure S4.d: COSY spectrum of compound 1

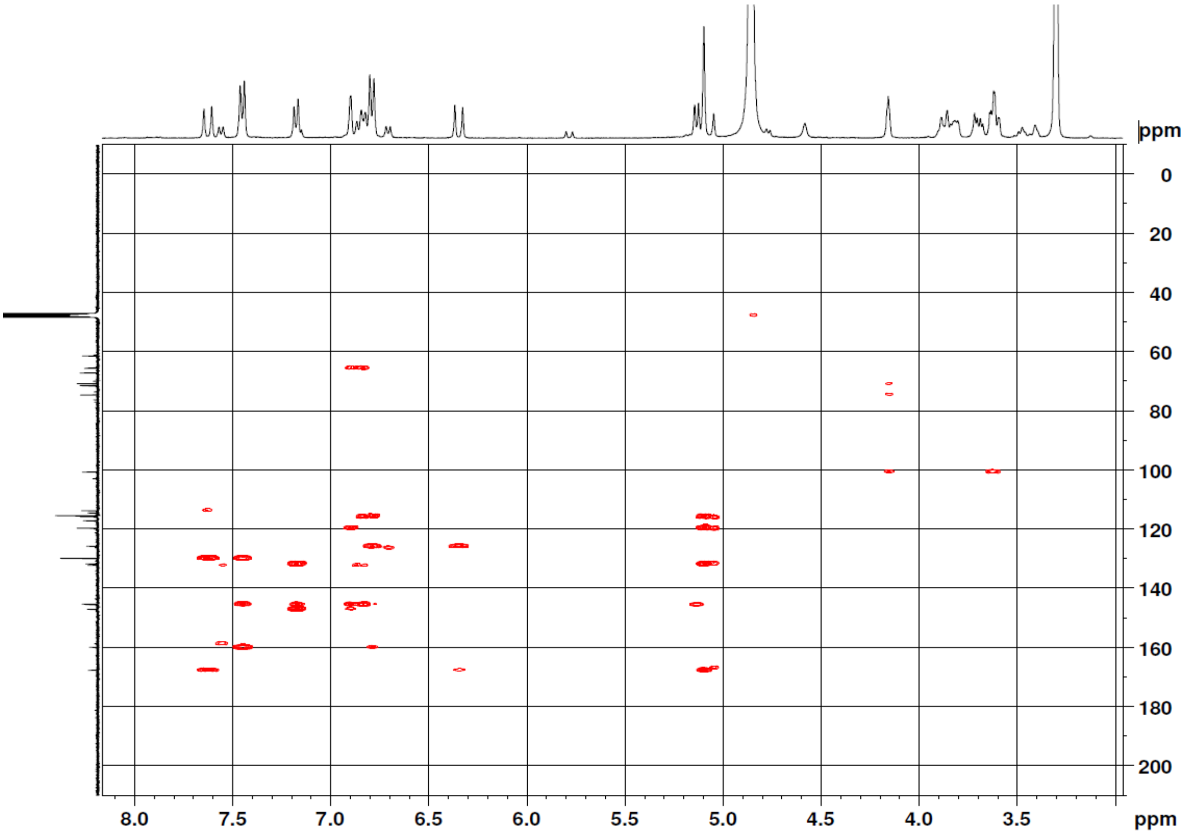

Figure S4.e: HMBC spectrum of compound **1**

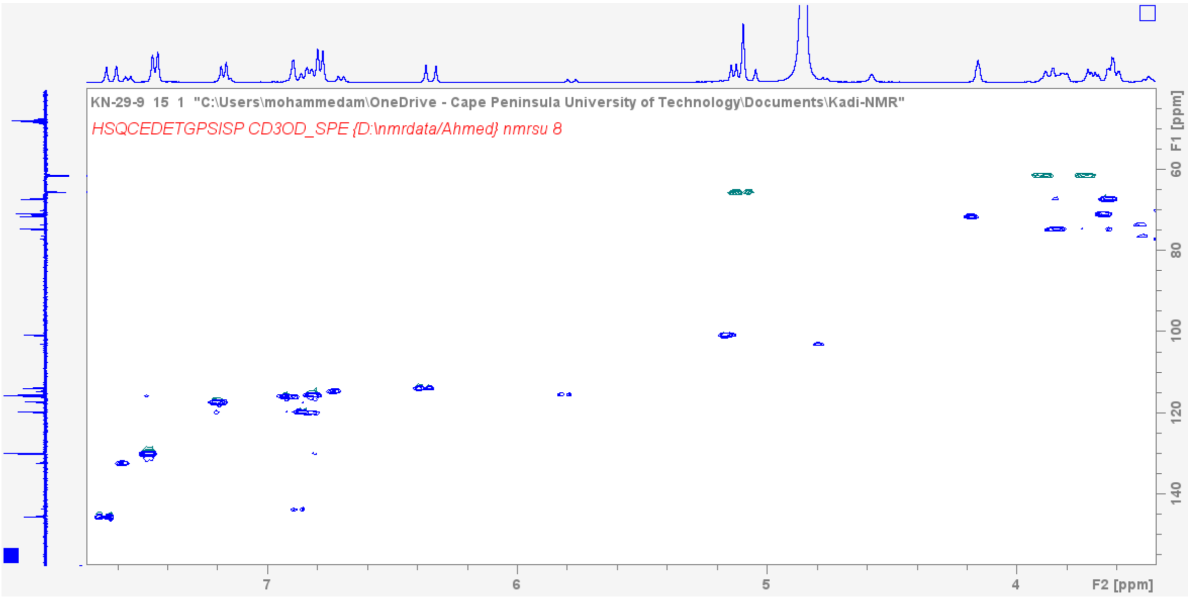

Figure S4.f: HSQC spectrum of compound **1**

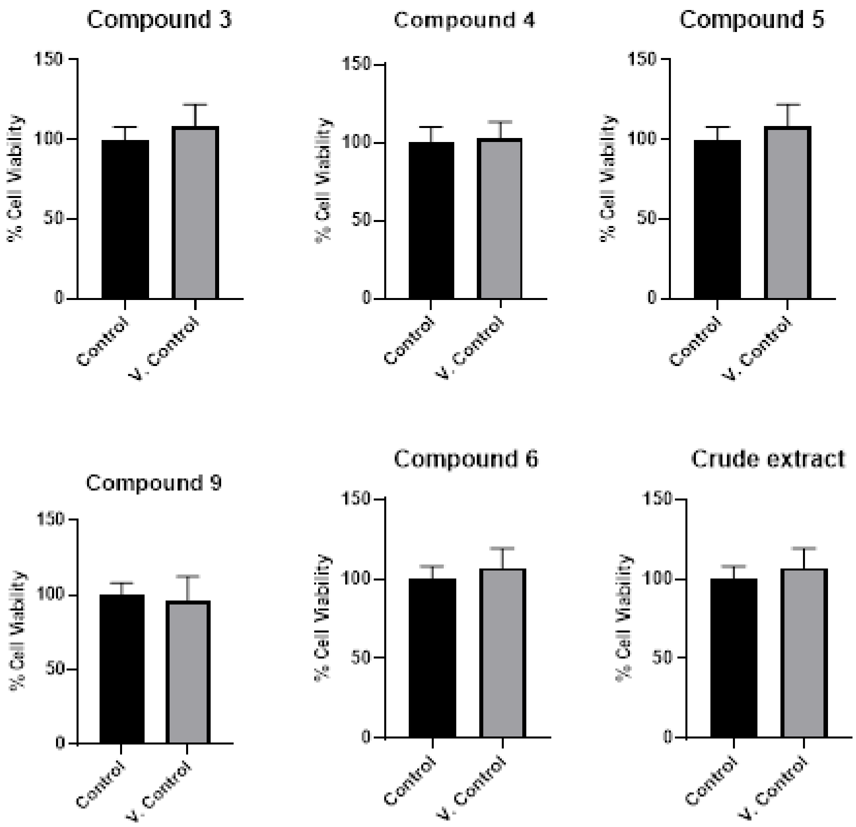

Figure S5: Comparative analysis: normal control vs vehicle control

Table S1: Active sites and Gridbox coordinates of the molecular docking simulations

	Active sites	Gridbox coordinates
**KEAP1**	E82, E83, E,78, E79, F83, Arg483, Arg415, Arg380, Asn382, Gln530, His436, Ser 555, Ser363, Ser508, Ser602, Tyr525, and Tyr334	Centre: x= 6.02888, y= 13.6967, z= 2.32762
		Size: X= 22.5578, Y= 21.743, Z= 23.9207
		Size: X= 31.3094, Y=32.2838, Z= 34.3109.
		Size: X= 39.0014, Y=33.9559, Z= 46.3329.

Table S2: Ligand Interaction Plot Analysis of P. Venus Compounds Bound to KEAP1

Compounds	Docking score (kcal/mol)	Hydrophobic interaction	Hydrogen bonds (length-Å)
**ML334**	-7.6	Cys434, Asp77, Leu84, Ile461, Arg483, Arg380, Gly433, His436, Phe478	Glu78 (2.92), Leu76 (2.90)
**RA889**	-6.0	Arg380, Arg483, Arg415, Asp77, Gln75, Glu79, His436, Ile461, Leu84, Gly480, Phe478	Glu78 (3.12), Leu76 (3.35)
**Compound 3**	-7.4	Glu79, Asp77, Ile461, Leu84 His436, Phe83, Phe478	Arg483 (3.18), Arg415 (3.15), Arg380 (3.04), Leu76 (2.71), Glu78 (3.21), Glu82 (2.87), Gln75 (2.86)
**Compound 4**	-7.0	Arg380, Arg483, Arg415, Asp77, Gln75, Glu79, His436, Ile461, Leu84	Glu78 (3.16), Leu76 (2.67)
**Compound 5**	-7.8	Arg 380, Leu84, His436, Ile461, Phe478	Arg483 (3.08), Arg415 (3.26), Asp77 (2.96), Leu76 (3.20), Glu79 (3.21), Glu78 (2.91/3.23), Gly433 (3.11)
**Compound 6**	-7.6	Gln75, Glu79, Asp77, Ile461, His436, Ser431, Phe478	Arg483 (2.81), Arg415 (3.08), Arg380 (2.76), Asp389 (3.30), Leu84 (3.05), Glu78 (3.26), Gly433 (2.70), Asn414 (3.17)
**Compound 9**	-6.0	Gly364, Gly462, Gly603, Gly464, Ala510, Ile461, Val604	Leu557 (2.87), Val463 (2.86/3.03)
